# First Cases of Dengue Fever Confirmed in the National Hospitals of Zinder and Niamey

**DOI:** 10.1155/crdi/6694128

**Published:** 2025-06-26

**Authors:** Doutchi Mahamadou, Adamou Lagare, Moussa Sahada, Adamou Bara Abdoul-Aziz, Mahaman Moustapha Lamine, Souleymane Adoum Fils, Bagnou Hamsatou, Hamidou Issa Hama, George Thomas, Ramatou Hamidou Lazoumar, Garba Abdoul Aziz, Adehossi Eric

**Affiliations:** ^1^Faculty of Medical Science, André Salifou University, Zinder, Niger; ^2^Zinder National Hospital, Zinder, Niger; ^3^Medical and Health Research Center (CERMES), Niamey, Niger; ^4^Faculty of Medical Science, Abdou Moumouni University, Niamey, Niger; ^5^Faculty of Sciences and Technology, André Salifou University, Zinder, Niger; ^6^Niamey National Hospital, Niamey, Niger

**Keywords:** arbovirus, dengue, hospitalization, Niger

## Abstract

Dengue is an emerging arbovirus infection caused by any of the four serotypes of dengue virus (DENV-1, DENV-2, DENV-3, and DENV-4) and transmitted via the bite of a mosquito from the genus Aedes. We hereby report seven cases of dengue hospitalized at the infectious and tropical disease departments of the National Hospitals of Zinder and Niamey from October 22, 2023, to December 05, 2023. All the cases presented fever, vomiting, algic, and hemorrhagic syndrome. Results from the complete blood count analysis revealed that all the patients had thrombocytopenia, six cases have leukopenia and two cases have anemia. Furthermore, one case of kidney failure was recorded. The case confirmation was conducted at the National Reference Laboratory for Arbovirus on blood samples using qualitative Real-Time Polymerase Chain Reaction (qRT-PCR). The technic is based on the amplification of any of the four serotypes of dengue virus nucleic acid using specific primers and probes as described by Wagner et al. All the cases recovered after hospitalization.

## 1. Introduction

Dengue is a vector-borne arbovirosis caused by an RNA virus of the *flavivirus* genus and transmitted by the bite of an arthropod mosquito of the genus Aedes [1, 2]. The infection is caused by any of the four dengue virus serotypes (DENV-1, DENV-2, DENV-3, and DENV-4) that are clinically closely related [3–5]. The disease is the most common arbovirosis in the world with about 390 million cases reported each year including 96 million symptomatic cases [1, 6]. Viral hemorrhagic fevers in general are now subject to global epidemiological surveillance by the World Health Organization (WHO), and considered as a public health emergency of international concern [2]. The incidence of dengue fever has gradually increased worldwide over the past 20 years, from 500,000 cases in 2000 to 5.2 million cases in 2019. A decrease in cases was observed between 2020 and 2021 due to the effect of the COVID-19 pandemic [7, 8]. However, since 2023, an upsurge of cases was observed with more than 5 million infections and the emergence of outbreaks in regions that were previously spared [9].

Dengue disease is endemic in more than 100 countries [10]. Since January 2023, a total of 180,634 cases including 20,736 confirmed and 462 deaths were reported in 14 African Union member states. The most affected countries were Burkina Faso, Ethiopia, Mali, Sudan, Chad, Sao Tome and Principe, Egypt, and Ivory Coast [11].

Niger Republic confirmed the first imported case of dengue due to DENV-3 in August 2022 from a patient returning from the Republic of Cuba [6]. Unfortunately, since September 2023, there has been an outbreak of dengue fever, with 148 suspected cases reported as of November 10, 2023.

We report seven cases of dengue hospitalized in the Infectious and Tropical Diseases Departments of the National Hospitals of Zinder and Niamey from October 22, 2023, to December 05, 2023.

## 2. Case No. 1

A 16-year-old female student with no known medical history was admitted to the Department of Infectious and Tropical Diseases at Zinder National Hospital on October 26, 2023. The reason for consultation was violent headaches, bloody diarrhea, easy-to-induce vomiting, bilateral anterior epistaxis, dry cough, rhinorrhea, and arthromyalgia evolving in a febrile context for 5 days. She had not reported any travel history.

Upon admission, the physical examination of the patient showed a consciousness, 99% pulse oxygen saturation, blood pressure of 125/80 mm Hg, heart rate of 85 beats/min, respiratory rate of 25 cycles/min, and a fever of 38.5°C. Also, the tourniquet test was positive. The biological analyses rendered the following results.

Blood count analysis showed a blood cell count at 7.700 elements/mm^3^, hemoglobin at 12.6 g/dL, and thrombocytopenia at 126,000/mm^3^. The C-reactive protein (CRP) was 12 mg/L. However, malaria test was negative. The International Normalized Ratio (INR) was 1.33, the activated headache time was 31.3 s, the prothrombin rate (TP) was 76.6%, and the transaminases were ALAT at 301 IU/L and ASAT at 406 IU/L. The GeneXpert test for tuberculosis (X-pert MTB) was negative. Blood sugar was 7.10 mmol/L. Renal function and blood ionogram were normal. Human immunodeficiency virus (HIV) serology, HBs antigen, and HCV antibodies were all normal. Arbovirus screening by qualitative Real-Time Polymerase Chain Reaction (qRT-PCR) revealed positive detection for dengue virus type 3 (Table 1). The patient had no complications during hospitalization. Therapeutically, the patient received injectable paracetamol, a hydration with salt serum. After 10 days, the blood count normalized with a platelet count of 187,000/mm^3^. The patient fully recovered after an 11-day period of hospitalization.

### 2.1. Case No. 2

A 22-year-old male student with no known medical history was admitted to the Infectious and Tropical Diseases Department of the Zinder National Hospital on October 26, 2023. The reason for consultation was moderate-intensity fronto-occipital headaches, accompanied by easy-to-induce vomiting, bilateral anterior epistaxis, muscle and joint pain, fever, chills, and sweating persisting for 6 days. The patient did not report any travel history. Upon admission, the physical examination found a conscious patient, with 98% pulse oxygen saturation, blood pressure of 125/80 mm Hg, heart rate of 101 beats/min, breathing rate of 21 cycles/min, and temperature of 38.8°C or 101.84°F.

At biological analysis, the blood cell count showed a leukopenia of 2600 elements/mm^3^, hemoglobin was 10.1 g/dL, and thrombocytopenia was 105,000/mm^3^. The malaria test was negative. Both kidney function and blood ionogram were normal. HIV serology and HBs antigen were also negative. Arbovirus screening by qRT-PCR revealed positive detection for dengue virus type 3 (Table 1). No signs of severity were noted during hospitalization. The patient was treated with injectable paracetamol and salt serum. After 3 days of hospitalization, the patient recovered.

### 2.2. Case No. 3

A 22-year-old woman, four-month pregnant and with no known pathological history, admitted to the infectious diseases department and tropical diseases department of the National Hospital of Zinder on October 28, 2023. The reason for consultation was violent headaches calmed by usual analgesics, retro-orbital pain, easy throwing vomiting, bilateral anterior epistaxis, gingivorragia, and arthromyalgia, all evolving in a febrile context for 4 days. She had not reported any travel history.

Upon admission, the physical examination found a conscious patient with conjunctivitis (pale mucous membranes and anicteric). The oxygen saturation pulse was 96%, the blood pressure at 95/58 mm Hg, a heart rate at 111 beats/min, a breathing rate of 25 cycles/min, and a temperature of 39.9°C.

Palpable and tender mandibular lymphadenopathy was observed. Liver and spleen examinations revealed no abnormalities.

The blood cell count showed leukopenia at 1400 elements/mm^3^, anemia at 6.7 g/dL, and thrombocytopenia at 36,000 elements/mm^3^. The malaria test was negative. The kidney function and blood ionogram were normal. HIV serology, HBs antigen, and HCV antibodies were negative. Arbovirus screening by qRT-PCR revealed positive detection for dengue virus type 3 (Table 1). The patient had no complications during hospitalization. The treatment was based on injectable paracetamol, salt serum, and a whole blood transfusion. At the control six days, the blood cell count showed leukocytes at 7000/mm^3^, hemoglobin at 9.1 d/dL, and platelets at 193,000/mm^3^. After 7 days of hospitalization, the patient recovered from the infection.

### 2.3. Case No. 4

A 44-year-old female teacher was hospitalized in the Infectious Diseases and Tropical Diseases Ward at the National Hospital of Zinder on December 05, 2023. The reason for consultation was violent headaches calmed by usual painkillers, back orbital pain, nausea, easy-to-induce throwing up, bilateral anterior epistaxis, gingivorragia, and arthromyalgia evolving in a febrile context for 4 days. She had no travel history for the past 3 months.

Upon admission, the physical examination found a conscious patient with a weight of 65.2 kg and a height of 1.65 m, well colored and anicteric conjunctivae and mucous membranes, a pulsed oxygen saturation at 87% (99% on oxygen at 4 L/min), blood pressure 120/80 mm Hg, heart rate 103 beats/min, respiratory rate 22 cycles/min, and temperature of 38°C.

Biologically, the blood cell counts showed leukopenia at 3700 elements/mm^3^, hemoglobin was 13.1 g/dL, and thrombocytopenia was 66,000/mm^3^. Renal function was impaired (uremia = 15.42 mmol/L and creatinine = 205 μmol/L and GFR 28.70 mL/min). The malaria thick drop test was positive. The blood ionogram was normal. HIV serology was negative. Arbovirus screening by qRT-PCR revealed positive detection for dengue virus type 3 (Table 1). The patient was administered injectable paracetamol, salt serum, and artesunate.

**Figure 1:**
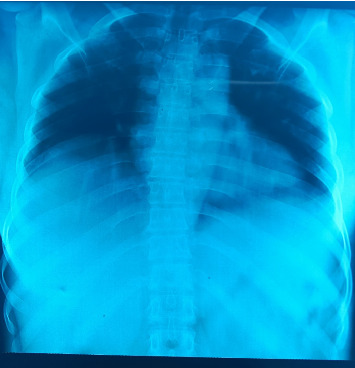
Chest x-ray revealing alveolar syndrome.

During hospitalization, the patient had presented alveolar pneumonia treated with amoxicillin (Figure 1). At control, the function was normalized with creatinine at 75 μmol/L and uremia at 5.6 mmol/L. The platelet rate had increased to 111,000 elements/mm^3^. Six days after hospitalization, the patient was discharged cured.

### 2.4. Case No. 5

A 24-year-old male student with no known medical history was admitted on November 11, 2023, to the Department of Infectious and Tropical Diseases at Zinder National Hospital. He was complaining about headache, dizziness, bloody diarrhea, postprandial vomiting, and bilateral epistaxis evolving in a feverish context for 4 days. No travel had been reported for the past 3 months.

Upon admission, the patient was found conscious and presents mucous and pale conjunctivae and signs of dehydration. Pulsed oxygen saturation was 98% at ambient air, blood pressure 91/62 mm Hg, heart rate 114 beats per minute, respiratory rate 27 cycles per minute, and temperature of 39.2°C.

Blood tests showed that leukopenia was at 3200 elements/mm^3^, hemoglobin 13.4 g/dL, and thrombocytopenia at 57,000/mm^3^. Blood ionogram and kidney function were both normal. Arbovirus screening by qRT-PCR revealed positive detection for dengue virus type 3 (Table 1). Clinical cure consisted with injectable paracetamol and salted serum. The control of blood cell count showed a persistence of leukopenia at 3600/mm^3^ and thrombocytopenia at 88,000/mm^3^. The patient had recovered after 6 days of hospitalization. The blood cell counts normalized 1 week after discharge.

### 2.5. Case No. 6

A 73-year-old woman retiree living in Niamey with known hypertension and treated with ramithiazide was admitted on October 30, 2023, at the infectious and tropical diseases department of the Niamey National Hospital. At consultation, complaints were noted for headaches, dizziness, and a state of asthenia all evolving for 3 days. No travel history had been reported in the past 3 months.

Upon admission, the patient was confused (Glasgow, 12), with moderate colored and anicteric mucous membranes and conjunctivae. Pulsed oxygen saturation was 97%, blood pressure was 90/60 mm Hg, heart rate was 124 beats per minute, respiratory rate was 28 cycles per minute, and temperature was 39, 6°C. Some purpuric stains on the thorax and the abdomen were noted.

The blood cell counts showed a leukopenia at 3360 elements/mm^3^, hemoglobin at 14.3 g/dL, and thrombocytopenia at 102,000/mm^3^. A cytolysis with ASAT at 107 μi/L and ALAT 26 μi/I was noted. The CRP was positive at 80.73 mg/L and urea at 7.30 μi/L. The creatinine and blood ionogram were normal. Arbovirus screening by qRT-PCR revealed positive detection for dengue virus type 3 (Table 1). Clinical cure consisted of injectable paracetamol and salted serum. The clinical evolution was good although it was prolonged by the persistence of asthenia. The control of blood cell counts revealed also a persistence of leukopenia at 3600/mm^3^ and thrombocytopenia at 112,000/mm^3^. The patient was discharged after 5 days of hospitalization. The BCC had normalized 1 week after the release.

### 2.6. Case No. 7

A 40-year-old woman living in Niamey with no known medical history was admitted to the Infectious and Tropical Diseases Unit of the Niamey National Hospital on October 22, 2023. The reason for consultation was moderate frontal headaches, muscle and joint pain, fever, chills, and sweating for 3 days in the aftermath of a pseudoinfluenza state for which she had undertaken oral malaria treatment with the association of arthemeter–lumefantrine. The patient had not reported travel history.

Upon admission, the physical examination found a conscious patient, with 98% pulse oxygen saturation, a blood pressure of 119/80 mm Hg, a heart rate of 112 beats/min, a respiratory rate of 22 cycles/min, and a temperature of 39.1°C.

Blood cell counts showed a leukopenia at 2800 elements/mm^3^, hemoglobin 10.5 g/dL, and thrombocytopenia 118,000/mm^3^. The malaria thick drop test was negative. Kidney function and blood ionogram were both normal. HIV serology and HBs antigen were also negative. Arbovirus screening by qRT-PCR revealed positive detection for dengue virus type 3 (Table 1). No signs of severity were noted during hospitalization. The patient was treated with injectable paracetamol and salted serum. After 3 days of hospitalization, the patient was cured and released.

## 3. Discussion

Dengue is a vector-borne disease, transmitted by a diurnal cosmopolitan mosquito known as *Aedes aegypti* [3, 12–14]. In Niger, the circulation of *Aedes aegypti* has already been reported by a previous study [15]. However, the detection of the virus has not been officially established, until a report of an imported dengue case in August 2022 [6]. For instance, the absence of travel history in all the seven patients presented in our study testifies support the hypothesis of local transmission of the virus. The propagation of the virus in Niger could be related to the epidemic of Burkina, Mali, Chad, and Nigeria [16–19]. From the epidemic trend in the West Africa region, the late notification of dengue cases in Niger could be due to the lack of awareness of the disease by clinicians and the lack of differential diagnosis with malaria and typhoid fever at the two National Hospitals [12]. Furthermore, the lack of rapid diagnostic testing in our context would also be a factor for under-reporting cases. Multiple cases of uncommon febrile illness were reported in Niamey, Niger, in mid-October 2023, leading to testing of fifteen samples, with 7 confirmed positive for mosquito borne dengue virus serotypes 1 and 3 [20]. However, there is no clinical description of the different cases of dengue fever observed in Niger.

In our study, the age of patients ranged from 16 to 73 years, with an average of 37.01 years and the female sex predominated. Previous studies suggest that age is related to dengue clinical symptoms, with older adults reporting fewer symptoms, higher age increasing the risk of clinical attack and severe dengue, and age-related differences in severity observed in both young infants and the elderly [21–24]. In the systematic meta-analysis review published by Guo et al. in 2017, they notified the predominance of dengue infection among a male [24]. This fact can be attributed to a combination of behavioral factors, such as increased outdoor exposure, and potential biological differences. Males may have higher exposure to mosquito bites due to outdoor activities and occupations that involve spending more time outside, where *Aedes* mosquitoes are prevalent [25].

Our results showed that all patients had nearly presented fever, vomiting, algic syndrome, and hemorrhagic syndrome. These constitute typical signs of dengue as reported by several studies [6, 21, 26]. Hemorrhagic sign was found minimal in this study population. This could be explained by the hypothesis that hemorrhagic dengue occurs mainly during secondary infections through the mechanism of immunological facilitation (*ADE: Antibody Dependent Enhancement*) [27–29].

Severe thrombocytopenia (platelet less than 20,000/mm^3^) was not found in this study. In 2016, Sondo et al. had a similar observation in Burkina [30]. According to Krishnamoorthy, severe thrombocytopenia (platelet less than 20,000/mm^3^) was statistically associated with mortality [31]. In others studies, while poor platelet recovery can lead to longer hospital stays, it does not necessarily correlate with increased mortality [32, 33].

Renal failure was found in only one patient and was the only complication recorded according to the 2009 WHO classification [34]. Contrary, Sondo in Burkina Faso found kidney damage in 20.2% [30]. However, lower frequencies were reported in Asia with a significant association with mortality [35, 36]. Dengue hemorrhagic fever patients with acute renal failure have a high fatality rate, emphasizing the need for clinicians to be alert for this potentially fatal complication [37].

Transaminase elevation was found in two cases although the rate had not reached 1000 UI/mL, which is considered as the criteria for severity. This liver damage could be mainly attributed to the replication phase of the virus in hepatocytes [38, 39]. Elevated transaminase levels can serve as early indicators of dengue infection and its severity, but their specificity as a sole predictor is limited due to significant overlap in values among different severity levels [37, 40, 41].

No deaths were recorded in this study. This result can be explained by the absence of signs of severity and comorbidities. Indeed dengue mortality is more frequent in patients with signs of severity such as hemorrhage and other comorbidities [21, 42]. Previous studies have shown that the main comorbidities associated with severe forms of dengue were diabetes and high blood pressure [26, 36, 43–45].

Therapeutic treatments for dengue fever in these patients mainly included injectable paracetamol to manage fever and pain and hydration with saline solutions to maintain hydration and electrolyte balance. In addition to these standard treatments, Ms. AI (Case no. 3) received a whole blood transfusion due to significant anemia and Mrs. MM (Case no. 4) was treated with artesunate for a concomitant malaria infection and with amoxicillin for alveolar pneumonia. No specific antiviral treatment for dengue fever was mentioned, with the main focus being on symptom management and possible complications during hospitalization. The primary treatment for dengue fever is symptomatic and supportive care, including fluid replacement and the use of analgesics to manage pain and fever [46]. Supportive care remains the main therapeutic strategy due to the lack of specific antiviral drugs approved for clinical use [47].

Our study provides a clinical overview of dengue fever cases in Niger, highlighting the most common symptoms including fever, vomiting, algic syndrome, and haemorrhagic syndrome. Notably, severe thrombocytopenia was absent, and renal failure was observed in only one patient as the sole complication. Transaminase elevation was mild and did not meet the criteria for severity. Importantly, no fatalities indicated a generally favorable outcome for dengue fever patients in this cohort. These findings reinforce the critical role of symptomatic management and supportive treatments in the absence of specific antiviral therapies for dengue fever.

## 4. Conclusion

Dengue is an emerging arbovirus that is generally benign. Signs of life-threatening severity should be systematically investigated. Improved surveillance passed through disposal of rapid diagnostic tests in order to improve early detection and manage clinical case. The fight against this disease involves vaccination and vector control. It is essential to monitor the circulation of the dengue virus in Niger to develop an algorithm that differentiates between dengue fever and malaria.

## Figures and Tables

**Table 1:** Breakdown of clinical and paraclinical signs.

	Effective (*n* = 7)	Percentage (%)
Sex		
Male	2	28.57
Female	5	71.43
Fever	7	100.00
Vomiting	5	71.42
Algic syndrome	7	100.00
Hémorrhagic syndrome	7	100.00
Thrombocytopenia	7	100.00
Leukopenia	6	85.71
Anemia	2	28.57
Hypercreatininemia	1	14.28
Dengue virus type 3 (qRT-PCR)	7	100.00

## Data Availability

Research data are not shared.

## References

[1] Bhatt S., Gething P. W., Brady O. J. (2013). The Global Distribution and Burden of Dengue. *Nature*.

[2] (2022). *ePILLY Trop 2022*.

[3] Harapan H., Michie A., Sasmono R. T., Imrie A. (2020). Dengue: A Minireview. *Viruses*.

[4] (2009). *Dengue: Guidelines for Diagnosis, Treatment, Prevention, and Control*.

[5] Sirisena P. D. N. N., Mahilkar S., Sharma C., Jain J., Sunil S. (2021). Concurrent Dengue Infections: Epidemiology & Clinical Implications. *Indian Journal of Medical Research*.

[6] Lagare A., Faye M., Fintan G. (2023). First Introduction of Dengue Virus Type 3 in Niger, 2022. *IJID Regions*.

[7] Chen Y., Li N., Lourenço J. (2022). Measuring the Effects of COVID-19-Related Disruption on Dengue Transmission in Southeast Asia and Latin America: a Statistical Modelling Study. *The Lancet Infectious Diseases*.

[8] Sasmono R. T., Santoso M. S. (2022). Movement Dynamics: Reduced Dengue Cases during the COVID-19 Pandemic. *The Lancet Infectious Diseases*.

[9] Who (2023). Dengue-Global Situation.

[10] Who (2023). Dengue and Severe Dengue.

[11] (2023). Dengue Fever in Africa: Two New Countries Affected (Togo and Cape Verde); Epidemics Particularly Active in Burkina Faso, Ethiopia and Mali.

[12] Simmons C. P., Farrar J. J., van Vinh Chau N., Wills B. (2012). Dengue. *New England Journal of Medicine*.

[13] Jansen C. C., Beebe N. W. (2010). The Dengue Vector Aedes aegypti: What Comes Next. *Microbes and Infection*.

[14] Paupy C., Delatte H., Bagny L., Corbel V., Fontenille D. (2009). Aedes albopictus, an Arbovirus Vector: From the Darkness to the Light. *Microbes and Infection*.

[15] Labbo R., Doumma A., Mahamadou I. (2019). Distribution and Relative Densities of Aedes aegypti in Niger. *Tropical*.

[16] Makadji M. (2023). *Dengue Epidemic in Bamako: Awareness Campaign Launched [Internet]*.

[17] (2023). Nigeria Reports an Epidemic of Dengue Fever in Sokoto State.

[18] (2023). Dengue-Tchad.

[19] Quotidien Sidwaya (2023). Dengue Epidemic in Burkina: At the Heart of the Epicentres [Internet].

[20] Amadou H. I., Moussa S., Arzika I. I. (2024). Emergence of Indigenous Dengue Fever, Niger, October 2023. *Emerging Infectious Diseases*.

[21] Amâncio F. F., Heringer T. P., Oliveira C. (2015). Clinical Profiles and Factors Associated with Death in Adults with Dengue Admitted to Intensive Care Units, Minas Gerais, Brazil. *PLoS One*.

[22] Hammond S. N., Balmaseda A., Solano S. (2005). Differences in Dengue Severity in Infants, Children, and Adults In A 3-Year Hospital-Based Study in Nicaragua. *The American Journal of Tropical Medicine and Hygiene*.

[23] Estofolete C. F., De Oliveira Mota M. T., Bernardes Terzian A. C. (2019). Unusual Clinical Manifestations of Dengue Disease – Real or Imagined?. *Acta Tropical*.

[24] Guo C., Zhou Z., Wen Z. (2017). Global Epidemiology of Dengue Outbreaks in 1990–2015: A Systematic Review and Meta-Analysis. *Frontiers in Cellular and Infection Microbiology*.

[25] Padonou G. G., Konkon A. K., Salako A. S. (2023). Distribution and Abundance of Aedes aegypti and Aedes albopictus (Diptera: Culicidae) in Benin, West Africa. *Tropical Medicine and Infectious Disease*.

[26] Lee I. K., Liu J. W., Yang K. D. (2008). Clinical and Laboratory Characteristics and Risk Factors for Fatality in Elderly Patients with Dengue Hemorrhagic Fever. *The American Journal of Tropical Medicine and Hygiene*.

[27] Madoff L. C., Fisman D. N., Kass-Hout T. (2011). A New Approach to Monitoring Dengue Activity. *PLoS Neglected Tropical Diseases*.

[28] Thomas L., Verlaeten O., Cabié A. (2008). Influence of the Dengue Serotype, Previous Dengue Infection, and Plasma Viral Load on Clinical Presentation and Outcome during a Dengue-2 and Dengue-4 Co-epidemic. *The American Journal of Tropical Medicine and Hygiene*.

[29] Deparis X., Maréchal V., Matheus S. (2009). Pathophysiological Mechanisms of Dengue: a Critical Review of Hypotheses. *Medecine*.

[30] Sondo K. A., Gnamou A., Diallo I. (2022). Descriptive Study of Dengue Complications during the 2016 Outbreak in Ouagadougou, Burkina Faso. *PAMJ-One Health [Internet]*.

[31] Krishnamoorthy S., Bhatt A. N., Mathew C. T., Ittyachen A. M. (2017). Hepatitis and Thrombocytopenia: Markers of Dengue Mortality. *Tropical Doctor*.

[32] Díaz-Quijano F. A., Villar-Centeno L. A., Martínez-Vega R. A. (2006). [Complications Associated to Severe Thrombocytopenia in Patients with Dengue]. *Revista Medica de Chile*.

[33] Tulara N. K. T. (2019). Dengue Fever. *Eastern Journal of Medical Sciences*.

[34] (2024). Dengue: Infections: ePOPI.

[35] Mallhi T. H., Khan A. H., Sarriff A., Adnan A. S., Khan Y. H., Jummaat F. (2016). Defining Acute Kidney Injury in Dengue Viral Infection by Conventional and Novel Classification Systems (AKIN and RIFLE): a Comparative Analysis. *Postgraduate Medical Journal*.

[36] Kuo M. C., Lu P. L., Chang J. M. (2008). Impact of Renal Failure on the Outcome of Dengue Viral Infection. *Clinical Journal of the American Society of Nephrology*.

[37] Lee I. K., Liu J. W., Yang K. D. (2009). Clinical Characteristics, Risk Factors, and Outcomes in Adults Experiencing Dengue Hemorrhagic Fever Complicated with Acute Renal Failure. *The American Journal of Tropical Medicine and Hygiene*.

[38] Alvarez M. E., Ramírez-Ronda C. H. (1985). Dengue and Hepatic Failure. *Americas Journal of Medicine*.

[39] Parkash O., Almas A., Jafri S. W., Hamid S., Akhtar J., Alishah H. (2010). Severity of Acute Hepatitis and its Outcome in Patients with Dengue Fever in a Tertiary Care Hospital Karachi, Pakistan (South Asia). *BMC Gastroenterology*.

[40] Mahmuduzzaman M., Chowdhury A. S., Ghosh D. K., Kabir I. M., Rahman M. A., Ali M. S. (2011). Serum Transaminase Level Changes in Dengue Fever and its Correlation with Disease Severity. *Mymensingh Medical Journal: MMJ*.

[41] Djossou F., Vesin G., Walter G. (2016). Incidence and Predictive Factors of Transaminase Elevation in Patients Consulting for Dengue Fever in Cayenne Hospital, French Guiana. *Transactions of the Royal Society of Tropical Medicine & Hygiene*.

[42] Toledo J., George L., Martinez E. (2016). Relevance of Non-communicable Comorbidities for the Development of the Severe Forms of Dengue: A Systematic Literature Review. *PLoS Neglected Tropical Diseases*.

[43] Lye D. C., Lee V. J., Sun Y., Leo Y. S. (2010). The Benign Nature of Acute Dengue Infection in Hospitalized Older Adults in Singapore. *International Journal of Infectious Diseases*.

[44] Low J. G. H., Ong A., Tan L. K. (2011). The Early Clinical Features of Dengue in Adults: Challenges for Early Clinical Diagnosis. *PLoS Neglected Tropical Diseases*.

[45] Saqib M. A. N., Rafique I., Bashir S., Salam A. A. (2014). A Retrospective Analysis of Dengue Fever Case Management and Frequency of Co-morbidities Associated with Deaths. *BMC Research Notes*.

[46] Wiwanitkit V. (2010). Dengue Fever: Diagnosis and Treatment. *Expert Review of Anti-infective Therapy*.

[47] Rajapakse S., Rodrigo C., Rajapakse A. (2012). Treatment of Dengue Fever. *Infection and Drug Resistance*.

